# Dimethylpolysulfides production as the major mechanism behind wheat fungal pathogen biocontrol, by *Arthrobacter* and *Microbacterium* actinomycetes

**DOI:** 10.1128/spectrum.05292-22

**Published:** 2023-10-06

**Authors:** Aline Ballot, Jeanne Dore, Marjolaine Rey, Guillaume Meiffren, Thierry Langin, Pierre Joly, Assia Dreux-Zigha, Ahmed Taibi, Claire Prigent-Combaret

**Affiliations:** 1 Laboratoire Ecologie Microbienne UMR 5557, Université Lyon 1, Villeurbanne, France; 2 Université Clermont Auvergne, INRAE, GDEC, Clermont-Ferrand, France; 3 Greencell, Saint-Beauzire, France; Pennsylvania State University, University Park, Pennsylvania, USA

**Keywords:** Bacteria-fungus interactions, wheat fungal pathogens, *Microbacterium*, *Arthrobacter*, volatile organic compounds (VOCs), biocontrol, mycotoxins

## Abstract

Volatile organic compounds (VOCs) produced by plant-associated microorganisms could serve as natural biocontrol compounds. In this work, we investigate the potential of wheat rhizosphere *Microbacterium* and *Arthrobacter* actinomycetes to inhibit the growth of major wheat phytopathogenic fungi, *Fusarium graminearum* and *Zymoseptoria tritici* via production of antifungal VOCs. A correlative analysis between fungal-growth inhibition versus bacterial volatilomic profiles identified four dimethylpolysulfide (DMPS) VOCs as the main active compounds behind their biocontrol potential. Subsequent inhibition assays reveal that DMTriS (dimethyltrisulfide) exhibits the strongest inhibition effects, then dimethyldisulfide (DMDiS). Further investigation focuses on the mechanisms behind *F. graminearum*-growth inhibition by the most active strain *Microbacterium* JM188 in dual culture. Surprisingly, nine interaction-induced VOCs, including two fungal sesquiterpenes, were exclusively detected in dual cultures, suggesting a complex interplay between microbial VOC production and sensing. More importantly, all JM188 VOCs, including antifungal DMPS, were less abundant in dual cultures, suggesting an uptake of bacterial VOCs by the fungus. Quantification of pure DMPS after confrontation with *F. graminearum* confirmed complete and partial uptake by the fungus of DMTriS and DMDiS VOCs, respectively, suggesting a potential link between the fungal uptake level of bacterial VOCs and their toxicity. Finally, we demonstrated that *F. graminearum* growth inhibition leads to a complete depletion of DON (deoxynivalenol) carcinogenic mycotoxins, highlighting a significant modulation of fungal metabolism. Collectively, these results pinpoint DMPS as broad-range fungal-inhibiting VOCs produced by rhizosphere *Microbacterium* and *Arthrobacter*, and emphasize the extensive VOC-mediated interplay between bacterial biocontrol agents and fungal pathogens.

**IMPORTANCE:**

As the management of wheat fungal diseases becomes increasingly challenging, the use of bacterial agents with biocontrol potential against the two major wheat phytopathogens, *Fusarium graminearum* and *Zymoseptoria tritici*, may prove to be an interesting alternative to conventional pest management. Here, we have shown that dimethylpolysulfide volatiles are ubiquitously and predominantly produced by wheat-associated *Microbacterium* and *Arthrobacter* actinomycetes, displaying antifungal activity against both pathogens. By limiting pathogen growth and DON virulence factor production, the use of such DMPS-producing strains as soil biocontrol inoculants could limit the supply of pathogen inocula in soil and plant residues, providing an attractive alternative to dimethyldisulfide fumigant, which has many non-targeted toxicities. Notably, this study demonstrates the importance of bacterial volatile organic compound uptake by inhibited *F. graminearum*, providing new insights for the study of volatiles-mediated toxicity mechanisms within bacteria-fungus signaling crosstalk.

## INTRODUCTION

Wheat, much like other plants, is affected by various pathogens that reduce both yield and grain quality (1). Two fungal pathogens, namely *Fusarium graminearum* and *Zymoseptoria tritici*, are responsible for major wheat diseases (2). *F. graminearum* is the main causal agent of several fusarium diseases, causing damages on roots and aerial parts as well as the production of deoxynivalenol (DON) mycotoxin, which result in reduction of grain size and quality (3), whereas *Z. tritici* is responsible for Septoria leaf blotch (4). *F. graminearum* exhibits a saprotrophic phase in soil and on plant residues (5, 6), making soil-borne inoculum of the pathogen a major source of primary infection (7). Wheat residues present on soil have been shown to support as well Z. *tritici* sexual reproduction (8, 9). It represents a key step in the Z. *tritici* life cycle, leading to genetic population diversification responsible for bypassing plant resistance (10, 11) and production of wind-dispersed ascospores (sexual spores) that initiate subsequent epidemics (12). Increased incidence and severity of both pathogens over the last three decades (13), along with several limitations of conventional crop-protection solutions [i.e., lack of adequate disease resistance among most commercial wheat cultivars (14), decreasing sensitivity to synthetic fungicide (15), pesticide use reduction, etc.] have favored active research for environmentally friendly biocontrol solutions. Among them, screening of biological control agents (BCAs) was done independently against these two pathogens in previous works (16 – 19). However, research and screening for efficient BCAs in controlling conjointly *F. graminearum* and *Z. tritici* were limited but could provide very efficient and valuable biopesticide.

BCAs can have biocontrol potentials through a variety of direct mechanisms involving the production of antifungal compounds and/or indirect mechanisms through nutrient competition or induction of plant defenses (i.e., induced systemic resistance) (20). The most described and applied BCAs strains are producers of soluble antifungal lipopeptides such as iturines and fengycins or extracellular hydrolytic enzymes and are mainly belonging to *Bacillus* and *Pseudomonas* genera (21, 22). The production of volatile organic compounds (VOCs) by soil- and plant-associated microorganisms has also long been recognized as a key feature and some bacterial VOCs have been documented for their effects on soil-borne pathogen fungi.

VOCs are defined as low-molecular-weight molecules (<300 Da) exhibiting high vapor pressure and low water solubility (23). They can disperse through the soil matrix, facilitating long-distance interactions among rhizosphere microorganisms and plants without direct contact between interacting partners (24). VOCs produced by species belonging to the *Bacillus*, *Burkholderia*, *Stenotrophomonas*, and *Pseudomonas* genera among others, have shown their ability to inhibit the growth of plant pathogens (25, 26). Reports of successful identification of such antifungal VOCs are limited, but a few, like dimethyl disulfide, dimethyl trisulfide, or acetoin have been reported to be responsible for antifungal activity (27 – 30). However, studies unraveling mechanisms of action of such VOCs on fungi are scarce.

Whereas both *Microbacterium* and *Arthrobacter* bacterial genera were showed to be prevalent in wheat rhizosphere microbiota (31), characterization of their biocontrol potential and underlying mechanisms of actions remains scarce in literature, especially with regard to their ability for VOCs production. Examples of previous studies showed that some *Microbacterium* and *Arthrobacter* strains were able to antagonize the three *Stemphylium lycopersici*, *Alternaria alternata*, and *Corynespora cassiicola* pathogens via VOCs production (32). However, only two studies have described VOCs-mediated antagonism by strains of *Arthrobacter* or *Microbacterium* genera with the identification of active VOCs and unraveling some of the involved molecular mechanism. Indeed, an *Arthrobacter agilis* strain was described as efficient in both inhibiting the growth of phytopathogenic fungi and in stimulating sorghum growth by producing the dimethylhexadecylamine VOC (33, 34). A *Microbacterium* strain was shown to have both plant-growth priming and antagonistic effects on the fungal root pathogen *Rhizoctonia solani* via dimethylpolysulfides (DMPS) VOCs production (35).

Considering that the infection cycles of *F. graminearum* and *Z. tritici* may begin with their survival on plant residues from the previous culture, biocontrol strategies targeting both pathogen populations in soils, using rhizosphere antagonizing BCA, could therefore hold significant relevance. In this study, we took interest in *Microbacterium* and *Arthrobacter* strains previously isolated from wheat rhizosphere soils and characterized for their efficiency in protecting wheat against Fusarium collar rot (31). Taking part in the general goal of developing BCA strains efficient at controlling both *F. graminearum* and *Z. tritici* populations, the first objective of this study was to characterize the *in vitro* antifungal activity via VOCs production of wheat rhizosphere strains belonging to *Arthrobacter* and *Microbacterium* genera over the two main fungal pathogens of wheat. As a second step, we used a correlation analysis between growth inhibition of both pathogens and bacterial volatilomic profiles to identify principal VOCs involved in the antifungal potential of these actinomycetes. To further unravel the underlying molecular mechanisms of identified antifungal VOCs, we then focused on the interaction between the most antagonist strain, *Microbacterium* JM188, *and F. graminearum*. Interplay between fungal and bacterial-produced VOCs during the biotic interaction was highlighted. The effect of bacterial VOCs on the production of DON mycotoxins by *F. graminearum* was analyzed, as well as their content dynamics in the dual-culture assay, which showed that the most effective antifungal VOCs are likely to be absorbed by the pathogen.

## RESULTS

### Mycelial growth and spore germination responses of *F. graminearum* and *Z. tritici* to VOCs produced by rhizobacteria

**Fig 1:**
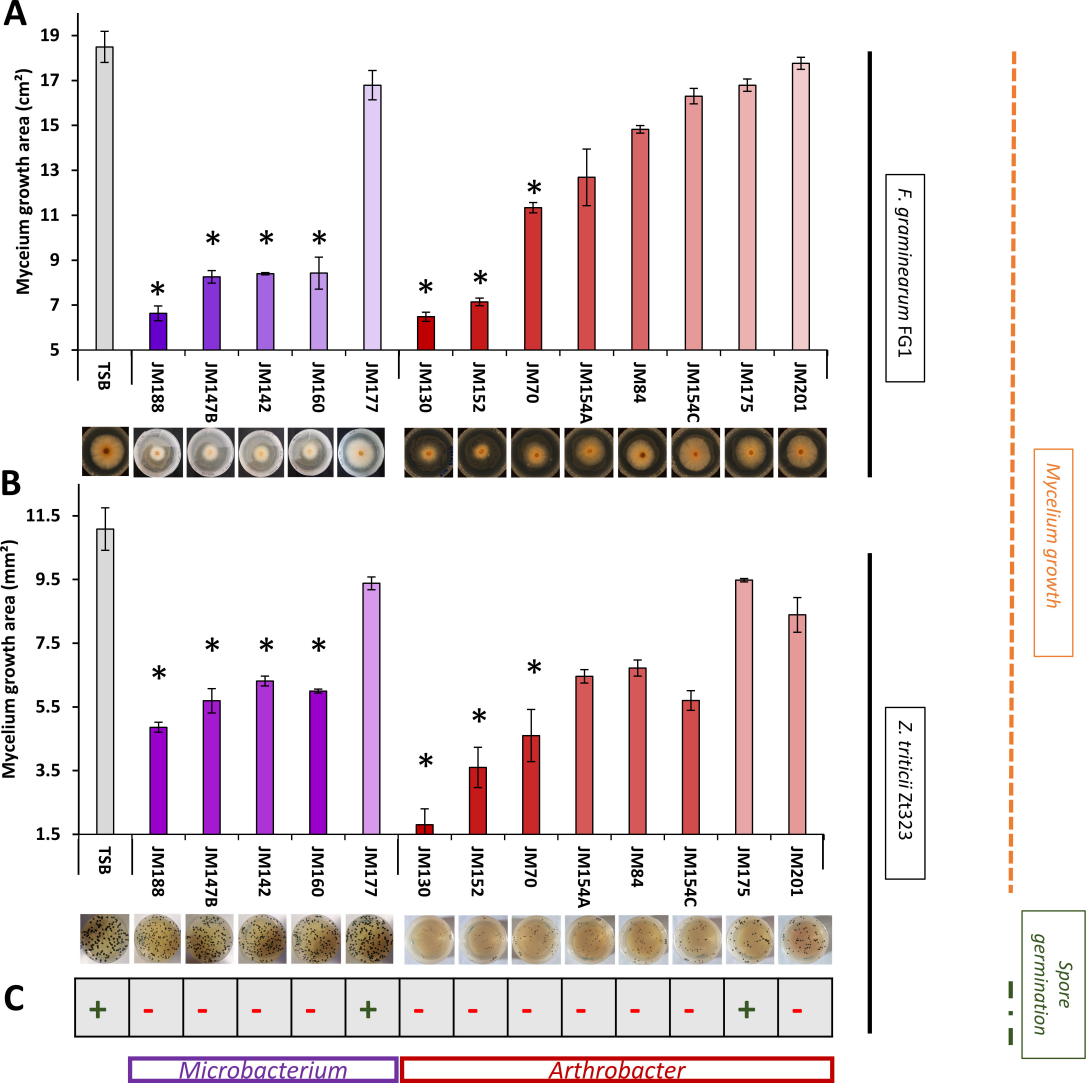
*In vitro* mycelial growth and spore germination inhibition effects by volatiles from *Microbacterium* and *Arthrobacter* rhizobacteria on *F. graminearum* Fg1 and *Z. tritici* IPO323. (**A**) Mycelial growth area of *F. graminearum* Fg1 (mean ± SE, *n* = 3), (**B**) Mycelial growth area of *Z. tritici* IPO323 (mean ± SE, *n* = 3), (**C**) Presence (+) or absence (−) of spore germination of *Z. tritici* IPO323. TSB: mycelial growth of pathogens exposed to sterile TSB medium (negative control). Asterisks indicate statistically significant differences based on pairwise comparisons between bacterial VOCs-exposed and TSB-exposed fungal cultures (Student’s *t* test, *P* < 0.05).

Five *Microbacterium* and nine *Arthrobacter* strains isolated from wheat rhizosphere soils were characterized for their growth inhibitory effects on the two major wheat pathogens *F. graminearum* (Fg1) and *Z. tritici* (IPO323), via VOCs. Mycelial growth areas were measured after 3 (Fg1) or 5 (IPO323) days of exposure to bacterial VOCs. VOCs from four out of five *Microbacterium* strains (JM188, JM160, JM147.B, and JM142) inhibited mycelium growth of both Fg1 (up to 64% reduction for the most active strain JM188) and IPO323 (up to 56% reduction for JM188)*,* compared to the TSB control (Fig. 1A and B). In addition, a complete inhibition of IPO323 spore germination was observed when confronted to VOCs of those four *Microbacterium* strains (Fig. 1C). Contrariwise, the *Microbacterium* JM177 strain showed no difference with the control on either Fg1 and IPO323 mycelium growth or spore germination.

Similarly, three groups of differential inhibition levels are observed over the set of *Arthrobacter* strains, with two strong inhibitory strains, JM130 and JM152, showing significant growth decreases of mycelium growth of both Fg1 (up to 63% reduction) and IPO323 (up to 83% reduction) (Fig. 1A and B). Intermediate results were observed for strain JM70 with 39% (Fg1) and 58% (IPO323) mycelium growth decrease. Poorly inhibitor *Arthrobacter* strains (JM175, JM201, JM154C, JM84, and JM154A) showed contrasted inhibition profiles between both pathogens with inhibition levels under 20% on Fg1 and between 13% and 48% on IPO323 mycelium. Nevertheless, a complete inhibition of IPO323 spore germination was observed for all *Arthrobacter* strains except JM175 (Fig. 1C). To determine the lethality of bacterial VOCs on *Z. tritici* spores, we checked if spore could germinate again when removing bacteria-inoculated plates after 10 days of VOCs exposure. Our results confirmed the sporicidal activity of bacterial VOCs, since no spore germination was observed.

### Volatilomic profiles of *Microbacterium* and *Arthrobacter* strains

**Fig 2:**
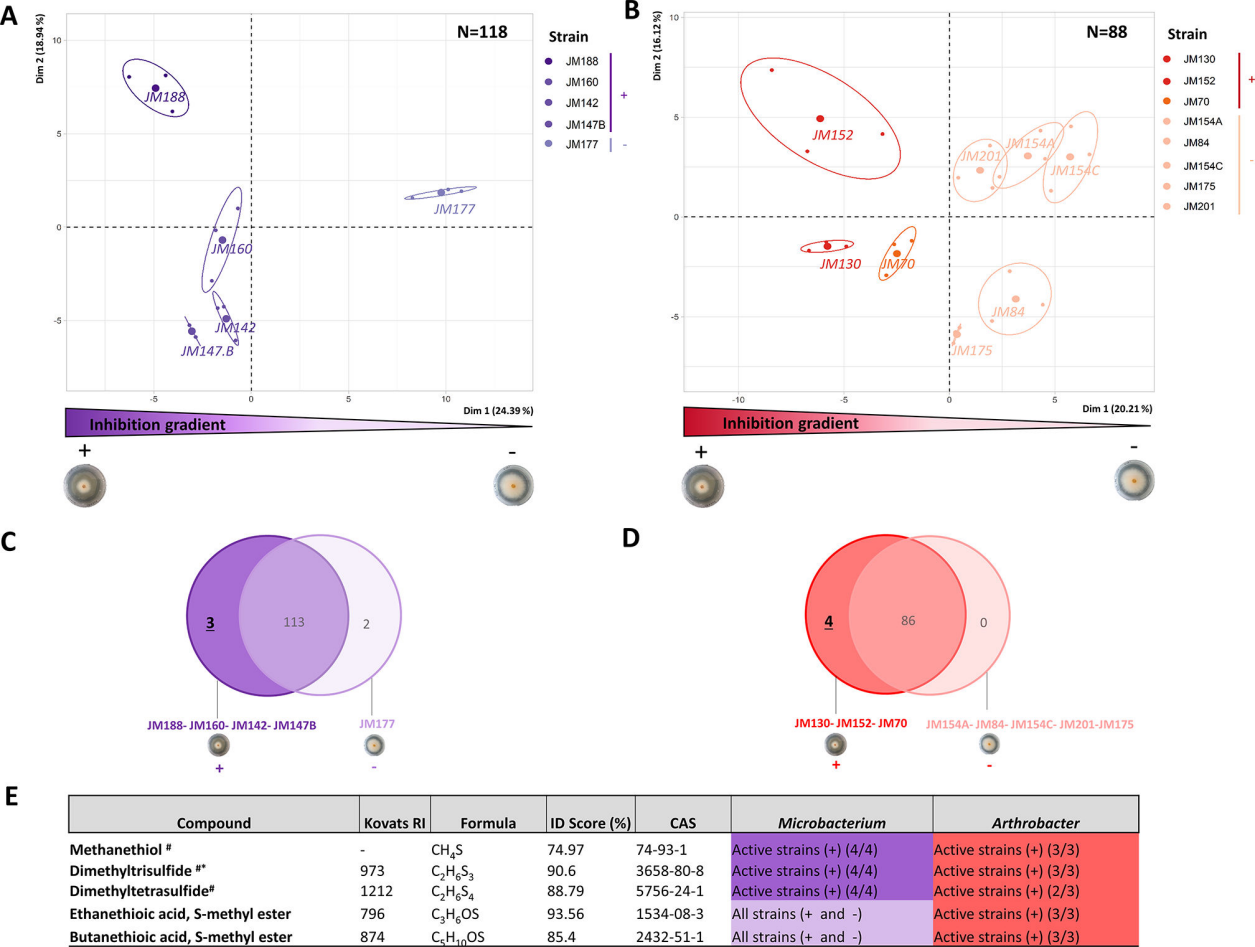
Comparison of volatilomic profiles across rhizobacteria strains with contrasted antifungal activities. (A and B) PCA were performed on integrated SPME/GC-MS peaks from *Microbacterium* (A) and *Arthrobacter* strains (B). Ellipses indicate the confidence interval at 0.95. (C and D) Venn diagram representing the number of unique and shared VOCs in each of the two *Microbacterium* groups of strains (C) and *Arthrobacter* groups of strains (D). (E) List of VOCs detected only in *Microbacterium* or *Arthrobacter* antifungal strains, annotated based on their mass spectra and calculated linear Kovats retention indices (RIs), and given with their molecular formula, identification (ID) score levels in % and CAS number. For each VOC, the number of strains producing it within the high antifungal (+) or low antifungal (−) group genera is indicated. ^#^VOCs present exclusively in high antifungal strains from both genera.

VOCs produced by the five *Microbacterium* and nine *Arthrobacter* strains, displaying contrasted antagonistic activities against Fg1 and IPO323 (Fig. 1), were analyzed through Headspace-Solid Phase MicroExtraction coupled with gas chromatography-mass spectrometry (GC-MS). Volatilomic profiles were composed of 118 and 89 different peaks for *Microbacterium* and *Arthrobacter* strains, respectively. Principal component analysis (PCA) on volatilomic profiles showed a separation of the *Microbacterium* (Fig. 2A) and *Arthrobacter* (Fig. 2B) isolates along the first two axes. For both bacterial genera, strains segregated according to their antifungal level (Fig. 1) on the first axes [i.e., 24.4% (Fig. 2A) and 20.2% (Fig. 2B) of total variability for *Microbacterium* and *Arthrobacter*, respectively].

The second axis (19% of total variability for *Microbacterium* strains, Fig. 2A) of PCA also discriminates the *Microbacterium* strains according to their inhibition potential with the most active strain M188 being separated from the non-active strain M177 and intermediate bioactive strains (i.e., M147.B, M160, and M142). Three main separated groups of *Microbacterium* strains were thus observed when considering the two axes (Fig. 2A). For the *Arthrobacter* strains, no relationship on axis 2 was observed between the strains and their antagonistic activity. Venn diagrams were constructed to compare VOCs produced by high antifungal (JM188, JM160, JM147B, and JM142 for *Microbacterium* and JM130, JM152, and JM70 for *Arthrobacter*) *versus* low antifungal strains (JM177 for *Microbacterium* and JM154A, JM84, JM154C, JM175, and JM201 for *Arthrobacter*) (Fig. 2C and D). Cross analysis (Fig. 2E) revealed that three VOCs are exclusively produced by high antifungal strains in both genera, i.e., *Microbacterium* strains JM188, JM147B, JM160, and JM142 and *Arthrobacter strains* JM70, JM130, and JM152. Mass analysis identified these three VOCs as methanethiol, dimethyltrisulfide (DMTriS), and dimethyltetrasulfide (DMTeS), which are all part of the DMPS group of VOCs. Other DMPS, such as dimethyldisulfide (DMDiS), were overproduced by high antifungal strains but were also produced by low antifungal strains.

### Correlation between DMPS production by *Microbacterium* and *Arthrobacter* strains and their antifungal activity

**Fig 3:**
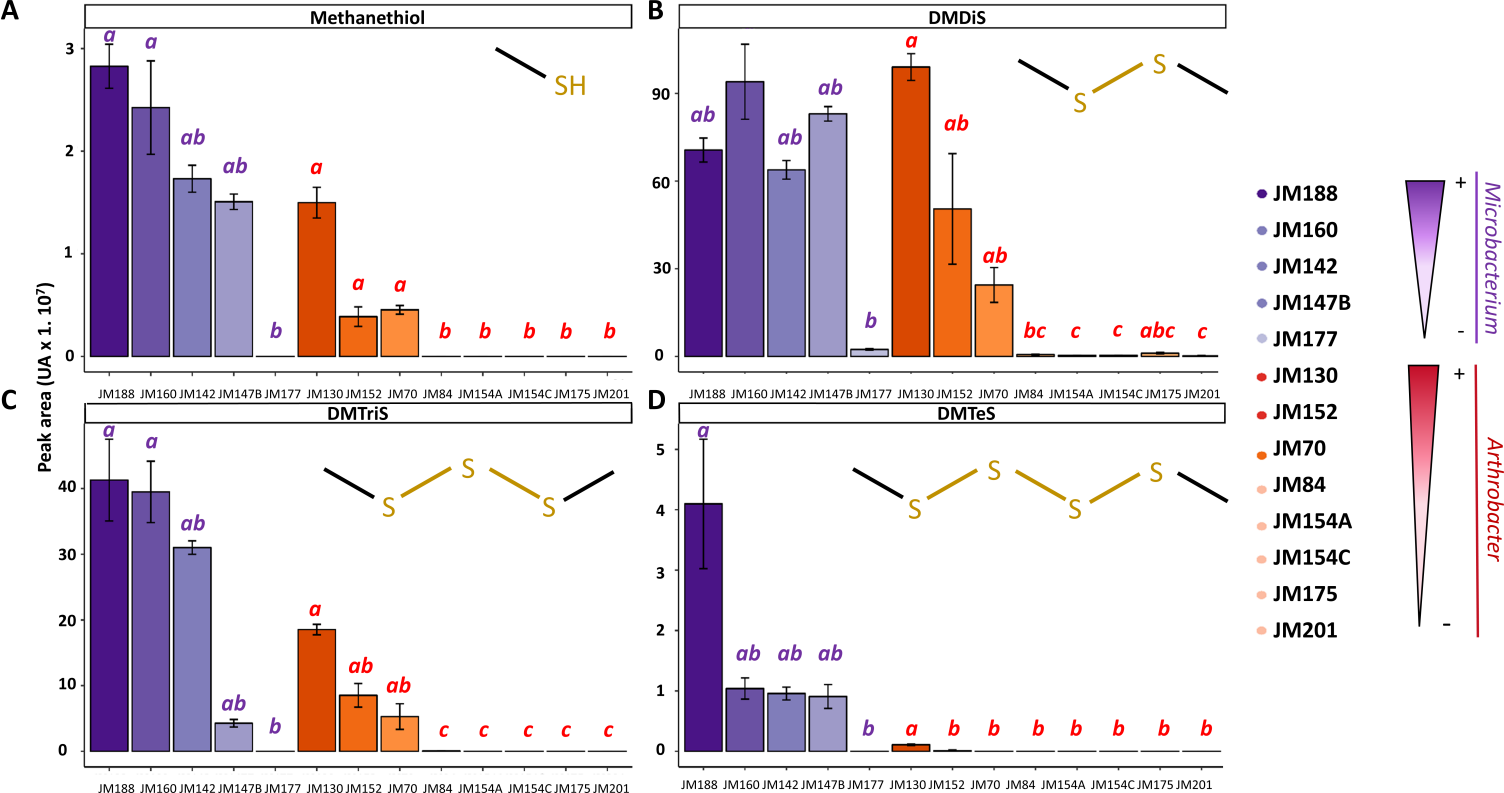
DMPS volatile peak areas detected in the headspace of the cultures of the five *Microbacterium* and nine *Arthrobacter* strains by SPME/GC-MS. (**A**) Peak areas of methanethiol, (**B**) peak areas of dimethyl disulfide (DMDiS), (**C**) peak areas of dimethyl trisulfide (DMTriS), and (**D**) peak areas of dimethyl tetrasulfide (DMTeS). Error bars represent mean ± SE, *n* = 3–4. Different letters show statistically significant differences (Kruskal-Wallis test, Dunn’s test post hoc test, *P* < 0.05) between strains of the same bacterial genus. Juxtaposed gradients indicate relative levels of fungal-growth inhibition of the respective strains.

To quantify DMPS production by the differentially active antifungal strains, area intensities of those VOCs were compared (Fig. 3). Significant differences were observed between low antifungal strains (i.e., *Microbacterium* JM177 and *Arthrobacter* JM175, JM201, JM154A, JM154C, and JM84) and high antifungal strains of both genera (i.e., *Microbacterium* JM188, JM160, JM142, and JM147B and *Arthrobacter* JM130, JM152, and JM70) in their production of all four detected DMPS (i.e., methanethiol, DMDiS, DMTriS, and DMTeS) (Fig. 3). Low antifungal strains did not produce any methanethiol (Fig. 3A), DMTriS (Fig. 3C) nor DMTeS (Fig. 3D).

In addition, some of these strains showed reduced DMDiS production (Fig. 3B) with measured areas of up to 100-fold lower than high antifungal strains (mean of 7.2 log10 [area] for the more DMDiS-producing strains JM175 and JM177 among poor antifungal strains, compared to a means of 8.89 for active strains of both genera). Moreover, a positive correlation between antifungal activity and the level of production of each DMPS VOC was clearly observed for high antifungal *Arthobacter* strains (JM130, JM152, and JM70; Fig. 3) (*R*² = 0.6–0.9; *P* < 0.05). A similar trend was observed for methanethiol, DMTriS, and DMTeS production by *Microbacterium* high antifungal strains (JM188, JM160, JM142, and JM147B; Fig. 3) (*R*² = 0.5–0.9; *P* < 0.05). In fact, the more the strains had an antifungal activity on both pathogens, the higher the peak areas of those compounds were. The most active strain JM188 was particularly distinct from the other high antifungal *Microbacterium* strains with a fourfold higher DMTeS production (Fig. 3D).

### Antifungal activity of DMDiS and DMTriS on fungal pathogen growth, as single compounds or mixtures

**Fig 4:**
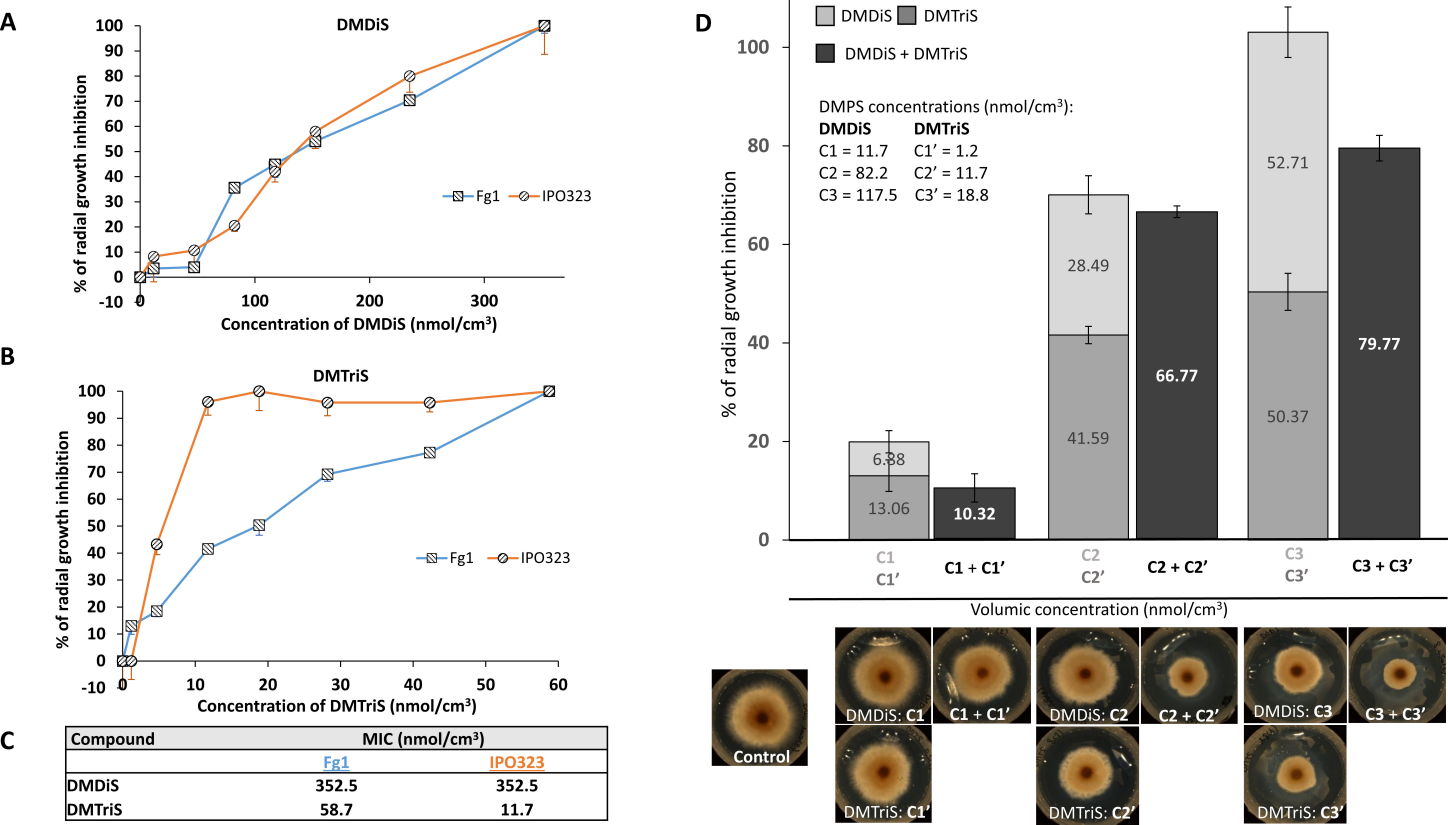
*In vitro* mycelial growth inhibition effects by pure DMDiS and DMTriS volatiles on *F. graminearum* Fg1 and *Z. tritici* IPO323. (**A**) Radial growth inhibition (%) on Fg1 and IPO323 mycelium by increasing concentrations of DMDiS ranging from 11.7 to 352.5 nmol/cm^3^. (**B**) Radial growth inhibition (%) on Fg1 and IPO323 mycelium by increasing concentrations of DMTriS ranging from 1.2 to 58.7 nmol/cm^3^. (**C**) Table indicating minimum inhibitory concentration (MIC) values of both DMPS on Fg1 and IPO323. (**D**) Radial growth inhibition (%) on Fg1 mycelium by three combinations of DMDiS and DMTriS concentrations: [C1 + C1′] = 11.7 + 1.2, [C2 + C2′] = 82.2 + 11.7, and [C3 + C3′] = 117.5 + 18.8 nmol/cm^3^ (dark gray), compared with added inhibitory effects of single DMDiS and DMTriS corresponding concentrations (stacked gray bars). Error bars represent mean − SE (**A AND B**) or mean ± SE (**D**), *n* = 3.

To determine if the DMPS overproduced by the most antifungal *Microbacterium* and *Arthrobacter* strains contribute to their antifungal activity, DMDiS and DMTriS were tested as single compounds and as mixtures for their effects on *F. graminearum* Fg1 and *Z. tritici* IPO32 mycelial growth (Fig. 4). Fungi were exposed to seven gradual concentrations of single compounds ranging from 11.7 to 352.5 nmol/cm^3^ for DMDiS (Fig. 4A), and from 1.2 to 58.7 nmol/cm^3^ for DMTriS (Fig. 4B). In addition, Fg1 was also exposed to mixtures of these compounds at concentrations of DMDiS and DMTriS ([DMDiS + DMTriS]): 11.7 + 1.2, 82.2 + 11.7, and 117.5 + 18.8 nmol/cm^3^ (Fig. 4D). Treatments were compared to control (DMSO solvent alone) and expressed as a relative growth inhibition (%). The results showed that DMDiS has a smaller inhibitory effect than DMTriS on mycelium growth of both fungi, with lower minimum inhibitory concentration (MIC) values of 58.7 nmol/cm^3^ for DMTriS versus 352.5 nmol/cm^3^ for DMDiS (Fg1), and 11.7 nmol/cm^3^ for DMTriS versus 352.5 nmol/cm^3^ for DMDiS (IPO323) (Fig. 4A through C). Moreover, the dynamics between fungal growth inhibition and DMPS concentrations were different for the two volatiles. The DMTriS curve showed a logarithmic relationship between compound concentration and fungal growth inhibition on both fungi, whereas we observed sigmoid-like relationship for DMDiS, on both fungi. DMDiS inhibitory levels were very similar between the two fungal strains, whereas DMTriS had a stronger inhibitory level on IPO323 strain with a fivefold lower MIC of 11.7 nmol/cm^3^ for IPO323 versus 58.7 nmol/cm^3^ for Fg1.

Inhibition percentages of the three DMPS mixtures, as compared to cumulated inhibition percentages of single compounds, showed no additive effects for two of the three mixtures: for the 11.7 + 1.2 nmol/cm^3^ mixture, the value of growth inhibition of 10.32% is lower than the sum of 6.38% and 13.06% inhibition obtained for DMDiS and DMTriS alone, respectively. Similarly, for the 117.5 + 18.8 nmol/cm^3^ mixture, the value of growth inhibition of 79.7% is lower than the sum of 50.4% and 53% inhibition obtained for DMDiS and DMTriS alone, respectively. However, an additive effect was observed with the mixture of 82.2 + 11.7 nmol/cm^3^ with a mean of 66.77% inhibition for the mixture versus 28.49% and 41.59% mean inhibition for DMDiS and DMTriS alone, respectively (adding up to 70% inhibition).

### Effect of the interactions between *F. graminearum* Fg1 and *Microbacterium* JM188 on volatilomic profiles

**Fig 5:**
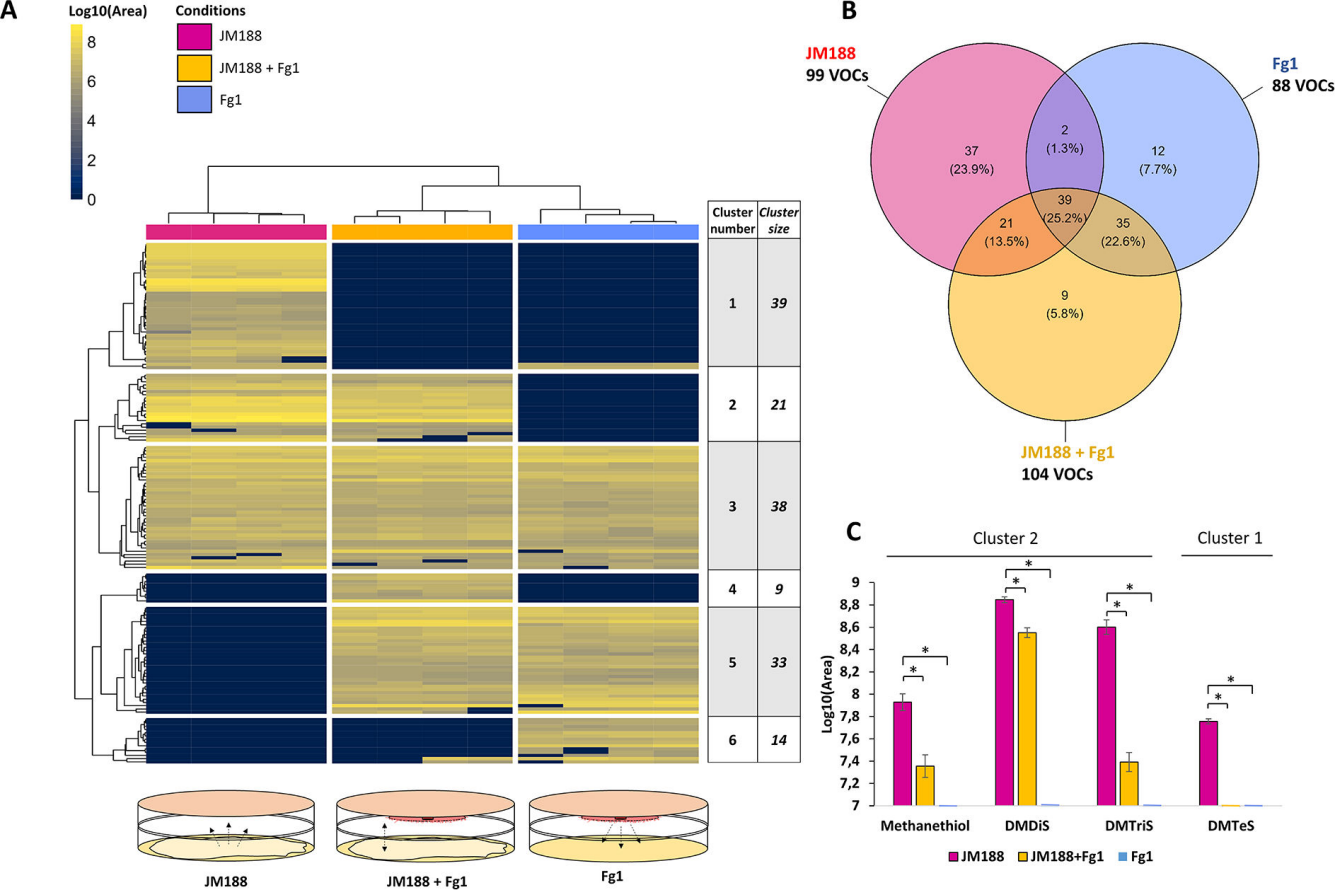
VOCs produced by JM188 and Fg1 alone or in confrontation. (**A**) Hierarchical clustering and heat map analyses of VOC profiles from JM188 and Fg1 control conditions and from confrontation conditions (JM188 + Fg1). Columns represent VOC profiles from four replicates per condition. Rows represent the peak area of each VOC (peak area in log10). The juxtaposed table indicates the cluster number from 1 to 6 and the number of VOCs included in each cluster. (**B**) Venn diagram representing shared and specific VOCs from the three conditions in number and percentage of total VOCs. (**C**) Quantification of DMPS peaks areas (mean ± SE, *n* = 4) for methanethiol, DMDiS, DMTriS, and DMTeS in JM188 and Fg1 controls, and in JM188 + Fg1 confrontation. Asterisks indicate statistically significant differences with the JM188 control condition (Student’s *t* test, *P* < 0.05).

To determine the effect of the biotic interaction between Fg1 and JM188 on the global volatilome, including specific DMPS, we analyzed the headspace volatile profile in dual cultures using a double-petri-dish setup. We then compared the two control conditions (Fg1 and JM188 alone) and the dual culture condition (JM188 + Fg1). Hierarchical cluster analysis and Venn diagram showed that 38 VOCs from cluster 3 are shared between the three conditions, representing 25% of the total number of VOCs detected in the three volatilomes (Fig. 5A and B), and between 38% and 44% of the number of VOCs in the JM188 and Fg1 control volatilomes, respectively.

The VOC profile of JM188 contained 37 VOCs from cluster 1 which were exclusive to JM188 culture volatilome, therefore absent from Fg1 control and from the confrontation culture (Fig. 5A and B). It represented 40% of total VOCs detected in the JM188 control condition. Twenty-one VOCs from cluster 2 were detected in both JM188 control and JM188 + Fg1 dual culture but not in the Fg1 control condition, so produced by the JM188 strain; 13 of them (62%) show statistically significant under-detection in dual culture as compared to JM188 (Table 1; Table S1). This means that 83% of VOCS exclusively produced by JM188 (clusters 1 and 2) were not detected or less detected when JM188 was confronted to Fg1. Those VOCs were putatively annotated as organosulfur compounds, alcohols, and alkenes (Table S1; Table 1).

**TABLE 1:** List of VOCs from JM188 and Fg1 control and JM188 + Fg1 confrontation conditions with identification hit match >88%

	Mean log10 (area)^*b*^			Statistical comparison^*c*^			Compound Identification^*d*^		
**RI^*a*^**	**Fg1**	**M188**	**M188 + Fg1**	**Fg1**	**M188**	**M188 + Fg1**	**VOC name**	**Formula**	**Cluster number^*e*^**
1031.93	0.00	7.91	0.00	a	b	a	1-Octanol	C_8_H_18_O	1
1178.67	0.00	7.05	0.00	a	b	a	2-Octene, 2,6-dimethyl	C_10_H_20_	1
1194.93	0.00	7.16	0.00	a	b	a	2-Propenoic acid, 6-methylheptyl ester	C_11_H_20_O_2_	1
1198.63	0.00	8.41	0.00	a	b	a	Tetrahydrogeraniol	C_10_H_22_O	1
**1230.65**	**0.00**	**7.60**	**0.00**	a	b	a	**Tetrasulfide, dimethyl^*f*^**	**C**_**2**_ **H**_**6**_ **S**_**4**_	**1**
1246.43	0.00	8.35	0.00	a	b	a	2-Propenoic acid, octyl ester	C_11_H_20_O_2_	1
1300.94	0.00	7.44	0.00	a	b	a	1-Tridecene	C_13_H_26_	1
**-**	**0.00**	**7.95**	**7.39**	**a**	**b**	**a**	**Methanethiol**	**CH**_**4**_ **S**	**2**
**730.25**	**0.00**	**8.85**	**8.56**	**a**	**c**	**b**	**Dimethyldisulfide**	**C**_**2**_ **H**_**6**_ **S**_**2**_	**2**
840.00	0.00	6.30	7.23	a	a	b	Ethyl 2-methylbutyrate	C_7_H_14_O_2_	2
**975.07**	**0.00**	**8.61**	**7.41**	a	b	a	**Dimethyl trisulfide**	**C**_**2**_ **H**_**6**_ **S**_**3**_	**2**
988.77	0.00	7.87	7.17	a	b	a	1-Heptanol, 6-methyl	C_8_H_18_O	2
992.78	0.00	7.33	6.60	a	b	a	1-Pentanol, 2-ethyl-4-methyl	C_8_H_18_O	2
1035.00	0.00	8.13	7.19	a	c	b	1-Octanol	C_8_H_18_O	2
1041.40	0.00	7.81	5.89	a	b	a	(s)-(+)−5-methyl-1-Heptanol	C_8_H_18_O	2
1207.39	0.00	6.98	7.36	a	b	c	2-Octene, 2,6-dimethyl	C_10_H_20_	2
716.30	7.07	6.79	7.81	a	a	b	Methyl-3-butanol-1	C_5_H_12_O	3
719.99	7.14	6.49	7.60	a	a	b	2-Methyl-1-butanol(isoamyl alcool)	C_5_H_12_O	3
866.65	7.46	7.44	7.33	a	a	a	Benzene, 1,3-dimethyl	C_8_H_10_	3
868.50	7.52	7.12	7.59	b	a	b	Benzene, 1,2-dimethyl	C_8_H_10_	3
1020.27	6.72	7.24	7.10	a	c	b	1-Heptanol, 3-methyl	C_8_H_18_O	3
1071.62	6.13	7.14	6.05	a	b	a	6-Dodecene, (z)	C_12_H_24_	3
1089.44	7.28	7.42	6.63	b	b	a	Alpha terpinolene	C_10_H_16_	3
1098.48	7.27	7.45	7.41	a	a	a	1-Decanol, 2-ethyl	C_12_H_26_O	3
1160.98	7.01	6.95	7.27	a	a	b	Nonane, 5-propyl	C_12_H_26_	3
1299.08	7.23	7.30	7.22	a	a	a	1-Octanol, 2-butyl	C_12_H_26_O	3
844.56	0.00	0.00	7.79	a	a	b	Ethylisovalerate	C_7_H_14_O_2_	4
1178.09	0.00	0.00	7.12	a	a	b	Acetic acid, octyl ester	C_10_H_20_O_2_	4
1486.83	0.00	0.00	6.09	a	a	b	Gamma muurolene	C_15_H_24_	4
1032.53	7.31	0.00	7.49	b	a	b	Cyclohexene, 1-methyl-4-(1-methylethenyl)-, (r)-	C_10_H_16_	5
1198.63	7.28	0.00	7.37	b	a	b	Dodecane	C_12_H_26_	5
1429.53	7.06	0.00	6.82	c	a	b	Alpha funebrene	C_15_H_24_	5
1443.85	7.76	0.00	8.02	b	a	c	Beta copaene	C_15_H_24_	5
1478.69	6.71	0.00	6.86	b	a	b	Alpha neocallitropsene	C_15_H_24_	5
1496.23	7.30	0.00	6.64	a	a	a	Germacrene-d	C_15_H_24_	5
1515.44	7.21	0.00	6.85	c	a	b	Beta curcumene	C_15_H_24_	5
1520.91	7.21	0.00	6.24	b	a	a	Beta himachalene	C_15_H_24_	5
1529.35	6.39	0.00	6.76	ab	a	b	Delta amorphene	C_15_H_24_	5
1535.12	7.96	0.00	6.11	b	a	a	(e)-Gamma-bisabolene	C_15_H_24_	5
1542.83	8.22	0.00	6.55	b	a	a	2-(1-Cyclopent-1-enyl-1-methylethyl) cyclopentanone	C_13_H_20_O	5
1454.41	7.11	0.00	0.00	b	a	a	*trans* caryophyllene	C_15_H_24_	6
1480.64	6.77	0.00	0.00	b	a	a	Alpha acoradiene	C_15_H_24_	6
1483.94	6.47	0.00	5.54	b	a	a	Beta acoradiene	C_15_H_2_	6
1492.78	7.67	0.00	0.00	b	a	a	Beta chamigrene	C_15_H_24_	6

^*a*^ Listed compounds correspond to compounds detected with peak areas at least two times as high as those of the blank control (medium only) and with identification match >88%. They represent 31% of all compound peaks detected. Retention Indices (RIs) were calculated.

^*b*^ Peak area mean in log10 are indicated.

^*c*^ Statistical differences between peak areas in the three conditions (N = 4) were evaluated with an ANOVA and Tukey’s HSD test (*P* < 0.05).

^*d*^ VOCs were putatively annotated by comparing their mass spectra and calculated linear RIs with those of online and in-house mass spectral libraries.

^*e*^ The cluster number from the heatmap hierarchical clustering (Fig. 5) of each detected VOCs is reported.

^*f*^ DMPS antifungal compounds are highlighted in bold.

In contrast, only 12 VOCs (cluster 6) were exclusively detected in Fg1 control condition, representing 13.6% of the total VOCs produced by Fg1. Thirty-five VOCs (clusters 5 and 6) were detected in both Fg1 control and JM188 + Fg1 dual culture but not in JM188 control condition, so produced by Fg1. They represent 40% of the total VOCs produced in Fg1 control conditions and among them, only five (15%) show statistically significant under-detection in dual culture as compared to Fg1 control (Table S1). Therefore, only 36% of the VOCs, exclusively produced by Fg1, were not detected or less detected when Fg1 is confronted to JM188. Those VOCs were putatively annotated mainly as belonging to sesquiterpene subclass (Table 1).

Cluster 4 showed that nine VOCs are specifically produced in JM188 + Fg1 confrontation condition (Fig. 5A and B). Two of them are putatively annotated as gamma-muurolene and 7-epi-cis-sesquisabinene hydrate (Table S1) from the sesquiterpene family.

DMPS compounds methanethiol, DMDiS and DMTriS are grouped in cluster 2 and the detailed analysis of their production levels (Fig. 5C) showed a significant decrease in JM188 + Fg1 as compared to JM188 control. This decrease is greater for DMTriS with a difference level of about 16-fold as compared to a 2-fold difference for methanethiol and DMDiS. DMTeS, in cluster 3, showed a mean production level of 7.8 log10(area) but was undetected in the confrontation condition.

### Inhibition of Fg1 DON mycotoxins by M188 volatiles

**Fig 6:**
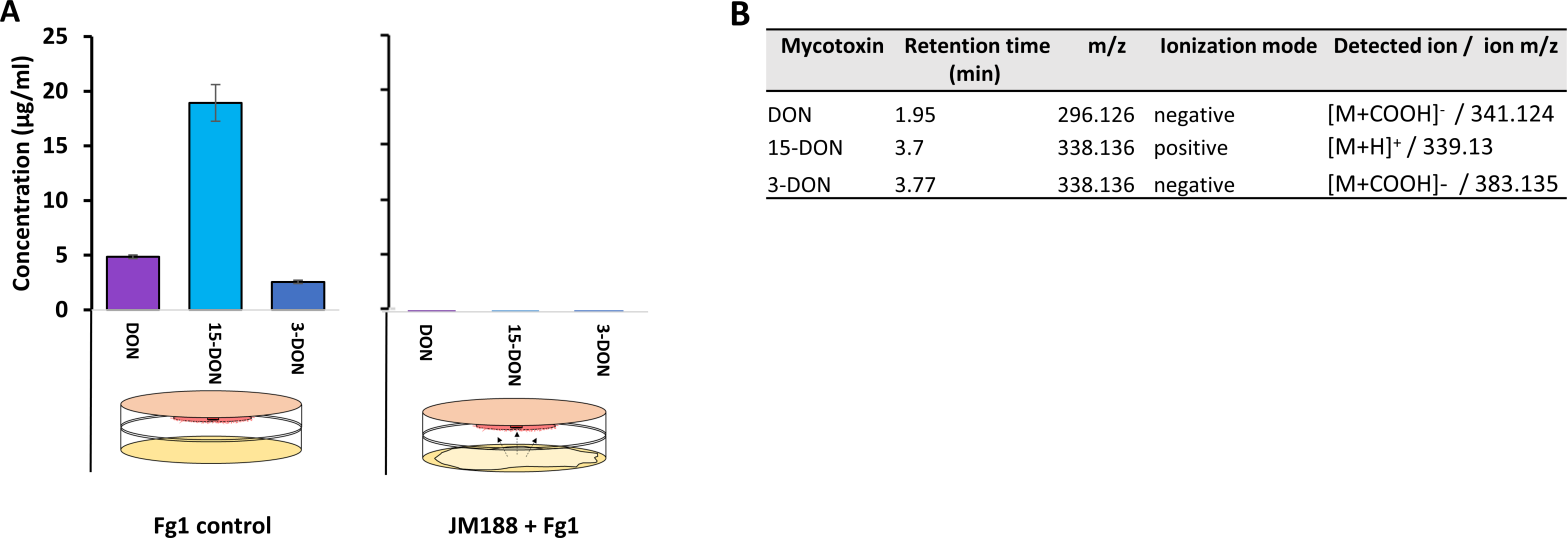
Mycotoxins concentrations in Fg1 metabolite crude extract of non-exposed (control) and M188 volatile exposed conditions. (**A**) DON and 3 of the 15 ADON mycotoxins concentrations in crude secondary metabolite extracts of Fg1. Bars represent means ± standard error (SE), *n* = 4. (**B**) Summary of mycotoxin detection parameters in LC-MS.

The same dual-culture samples, previously compared in terms of volatile production, were used for secondary metabolites extraction from Fg1 PDA-culture. Detection of DON mycotoxin and of two acetyl derivates, 3-acetyldeoxynivalenol (3-ADON) and 15-acetyldeoxynivalenol (15-ADON), was done on 10 mg/mL of metabolite extracts by comparison with standards in LC-MS (Fig. 6B; Fig. S2), allowing calculation of DON concentrations in both conditions of dual cultures (i.e., in presence and absence of JM188 volatiles).

All three trichothecene B mycotoxins were detected and measured in the control condition of Fg1 culture (without contact with any bacteria) (Fig. 6A). The 15-ADON mycotoxin was the most produced with a mean concentration of 19 µg per mL of extract, as compared to DON (5 µg/mL) and 3-ADON (2.5 µg/mL). By contrast, none of the three mycotoxins were detected when Fg1 was in contact with VOCs from JM188, showing that those mycotoxins are either not produced or less produced in dual cultures (i.e.*,* below the detection limit of 500 ng/mL).

### Quantification of DMDiS and DMTriS in the headspace of double-petri-dish setup in the absence or presence of Fg1 growing mycelium

**Fig 7:**
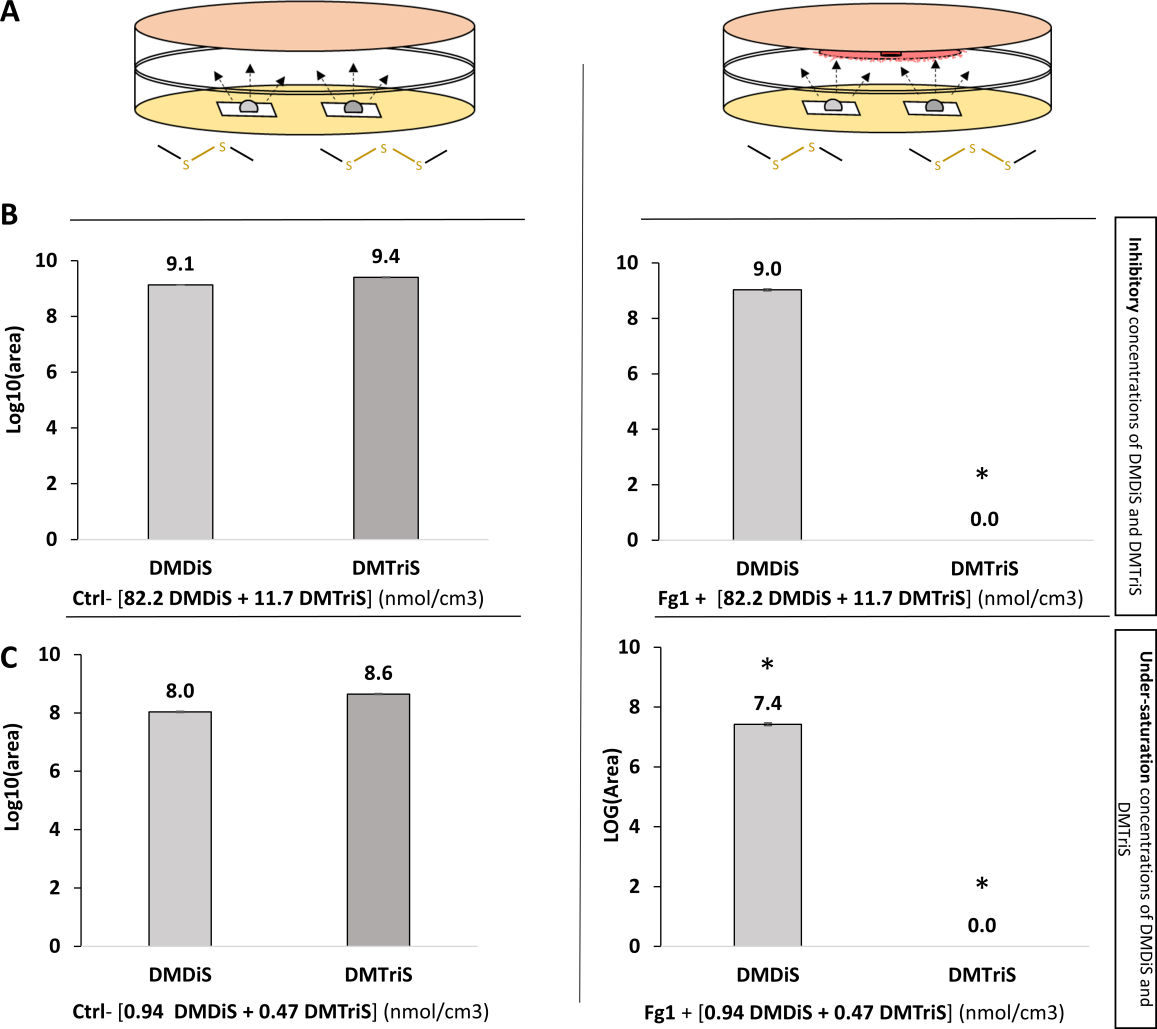
Quantification by SPME/GC-MS of DMDiS, DMTriS after 3 days in petri headspace, in presence or absence of Fg1. (**A**) Experimental setup for exposing Fg1 mycelium to DMPS volatile synthetic compounds *in vitro*. (**B**) Quantification of DMPS peak areas (mean ± SE, *n* = 4) in petri dishes for PDA control and Fg1 mycelium culture conditions. Equivalents of 82.2 and 11.7 nmol/cm^3^ of DMDiS and DMTriS were added in petri dish assemblages at J0 (each concentration corresponds to inhibitory concentration of Fg1 mycelial growth). Asterisks indicate statistically significant differences based on pairwise comparisons between control and Fg1 culture conditions (Wilcoxon test, *P* < 0.05). (**C**) Quantification of DMPS peak areas (mean ± SE, *n* = 4) in petri dishes for PDA control and Fg1 mycelium culture conditions. Equivalents of 0.94 and 0.47 nmol/cm^3^ of DMDiS and DMTriS were added in petri dish assemblages at J0. They are both under inhibitory concentrations of Fg1 mycelial growth and under saturation concentrations in GC-MS.

To test the effect of Fg1 mycelium culture on DMPS quantities, two different combinations of both DMPS were tested within the double-petri-dish setup (Fig. 7A). Inhibitory concentrations of mixture of DMPS allowed us to quantify DMPS accumulation in the presence of Fg1 at biologically relevant concentrations, whereas the quantification of DMPS under-saturation concentrations allowed their quantification in the linear phase of area-concentration relationships in SPME-GC (see Fig. S3). Contact of Fg1 with the inhibitory concentrations of 82.2 + 11.7 nmol/cm^3^ of DMDiS and DMTriS resulted in a complete depletion or lack of detection of DMTriS after 3 days (Fig. 7B). On the contrary, DMTriS was detected, up to a 9.4 log10(area) in the absence of Fg1 in the control condition (PDA medium only). DMDiS was detected in both conditions at a statistically similar level of 9 log10(area). Contact of Fg1 with DMPS in under-saturation concentrations (0.94 + 0.47 nmol/cm^3^) of DMDiS and DMTriS (Fig. 7C) resulted in the same observations of a complete lack of detection of DMTriS, which could be detected in the control condition at 8.6 log10(area). DMDiS was still detected in the presence of Fg1 as in the control, but in a significantly lower quantity of 7.4 log10(area) versus 8 log10(area) in the control.

## DISCUSSION

Members of the *Microbacterium* and *Arthrobacter* genera are widespread in the rhizosphere (31); however, as with most non-filamentous actinomycetes, there is little information on their VOC-mediated effects on saprophytic plant pathogens. In this study, the antifungal effects of VOCs from wheat rhizosphere strains on *Z. tritici* and *F. graminearum* wheat pathogens were described, and the discriminant volatiles associated with strong VOCs-mediated fungal growth inhibition uncovered. The antifungal activity of the identified bacterial VOCs on pathogens was also confirmed using available synthetic DMPS VOCs, namely, DMDis and DMTriS. In addition, our data showed that bacterial antifungal VOCs were depleted during bacterial-fungal confrontation with *F. graminearum* and demonstrated a positive correlation between the antifungal potential of one DMPS and the level of depletion, suggesting that highly active VOCs are absorbed by *F. graminearum*.

To date, most studies on the antifungal potential of bacteria have been described mainly on strains belonging to the bacterial genera *Bacillus* (36 – 39) and *Pseudomonas* (40, 41). Here, we demonstrated that *Microbacterium* and *Arthrobacter* antagonistic potential against wheat pathogens was correlated with the production of the DMPS VOCs methanethiol, DMDiS, DMTriS, and DMTeS. These compounds are maybe not the only actors of antifungal effects, but their high prevalence in bioactive strains suggests they might be the main contributors. Production of those DMPS and in particular DMDiS and DMTriS volatiles is widespread among living organisms including microorganisms and has been reported to be produced by rhizobacteria belonging to different genera such as *Bacillus* (42), *Pseudomonas* (41, 43, 44), *Stenotrophomonas* (43), and *Microbacterium* (45). Although there is only one previous study making the statistical correlation between antifungal activity and high production of DMPS (46), a number of accumulating studies have reported a link between abundant bacterial production of DMPS and antifungal activity against fungal pathogens infecting crops such as *Botrytis cinerea* (43) and *Rhizoctonia solani* (35). Collectively, these results further prove the role of bacterial DMPS volatile organic compounds in controlling fungal soil pathogens and prevalence of their production by rhizobacteria. Production of these DMPS is known to be derived from cysteine and methionine amino acids (47), via degradation enzymes like methionine *γ*-lyase (48). Consistently, sequences encoding a methionine γ-lyase (*mgL),* two cystathionine γ-lyases (*mccB1/mccB2*), and a cystathionine β-lyase (*cbL*) were found in the genome of the DMPS-producing active *Microbacterium* strain JM147B (data not shown). Moreover, we demonstrated that DMPS are produced by JM147B in minimum medium only when it is supplemented with methionine and cysteine (data not shown), confirming the sulfur-amino acid catabolism as the main catabolic pathway to DMPS production. This suggests that rhizosphere plant exudates may be a substantial provider of DMPS sulfur-amino acid precursors, which could explain the prevalence of DMPS-producing strains in rhizosphere soils.

Study of individual DMPS antifungal potential is limited by their availability as pure compounds. However, our results from *in vitro* confrontation confirmed the toxicity effect of DMDiS and DMTriS on both *Fusarium* and *Zymoseptoria* reference strains. These results are consistent with previous *in vitro* assays and *in planta* results showing antifungal, and nematicide action of pure DMDiS and DMTriS (49, 50), with DMDiS being studied and used as soil fumigant to protect crops (51 – 53). A study showed efficient soil disinfection over *Fusarium oxysporum* by DMDiS (54). Moreover, our results show that DMTriS has a stronger toxicity than DMDiS showing about 6- to 30-fold lower inhibitory concentrations on respectively *F. graminearum* and *Z. tritici*, consistent with a 10-fold ratio described in study related to *Penicillium italicum* inhibition by DMPS (40). This suggests a broad range of toxic effects of DMDiS and DMTriS on fungi with non-specific underlying mechanisms, which is consistent with results suggesting alteration of cell wall and membrane (50, 55). Effects of mixtures of the two DMDiS and DMTriS were not previously tested for their antifungal effects but only for their plant growth-promoting potential (45) or to test the attractiveness of DMDiS and DMTriS to saprophilous flies (56). In our study, we showed that for the three similar DMDiS/DMTriS concentration ratios tested, the effect of mixtures depends on levels of concentrations with additive effects for 82.2 + 11.7 nmol/cm^3^ of DMDiS and DMTriS and antagonistic effects for 117.5 + 18.8 nmol/cm^3^. However, no synergistic effect was observed on chosen concentrations. This suggests similar mechanisms of toxicity by these two VOCs on fungi, but this work should be extended to more concentration ratios and levels, as well as more pathogenic fungi in order to conclude on the type of effects of DMPS mixtures.

It is noteworthy that in addition to reducing Fg1 mycelium growth, we measured an antimycotoxic effect of JM188 strain volatiles on DON and ADON mycotoxins production. This result supports the potential of *Microbacterium* strain JM188 to be used as a biocontrol strain for surface-spikelet treatment at anthesis to reduce fusarium head blight incidence, alternatively or in addition to a soil inoculation. There is accumulated evidence in literature showing that the production of fungal secondary metabolites, DON and ADON in particular, could be an element of the general stress response in *F. graminearum*. Abiotic signals like hydric stress, temperature, pH (57), or the application of biosynthetic antifungal molecules (58) along with biotic signals associated with the inoculation of antifungal BCAs (59) were shown to modulate fungal mycotoxin biosynthesis. However, these effects are highly variable and complex, and a reduced growth of *Fusarium* does not necessarily mean a decrease of mycotoxin content (60). Among BCAs, we have examples of strains efficiently reducing *F. graminearum* mycotoxin production (59, 61) while others reduce mycelium growth but enhance mycotoxin production: for example, co-cultures of a *Pediococcus pentosaceus* strain with pathogenic *Fusarium verticillioides* led to both a reduction in pathogen’s growth and an increase in fumonisin production (62).

It is recognized that VOCs act as communication signals in soil microbiota and rhizosphere. However, the volatilomic responses of bacteria and fungi in a context of VOCs mediated contact in dual-culture remain rather unknown. A significant finding from our volatilomic analysis of VOCs dual-culture confronting *Microbacterium* JM188 antifungal strain and *F. graminearum* Fg1 mycelium is that volatile profiles of both the bacteria and the fungus are highly modulated, with lower quantity or even no detection of several VOCs in dual-culture as compared to their respective monocultures. This suggests either a strong downregulation of bacterial and fungal VOCs production as an interaction outcome, and/or a mutual cross-absorption/adsorption of VOCs produced by co-cultured partners. Despite *Microbacterium* growth not being affected in the presence of the fungal pathogen, the biggest differences were observed in its volatilome, with almost all *Microbacterium* VOCs being significantly measured in lower quantity in dual-culture as compared to monoculture. In contrast, while *Fusarium* growth was highly reduced in dual cultures (by approx. 60%), only about 36% of its VOCs were detected in lower amounts in dual-culture. Only three other studies have tackled the subject of volatile and/or non-volatile metabolomes in the context of VOCs-confrontation between two microbial agents (63 – 65); all of them reported modulation of VOCs detection comparing dual and monocultures- but not necessarily down-regulation along with production of confrontation-induced specific VOCs.

Among bacterial VOCs detected in lower amounts in dual cultures are the four antifungal DMPS. Another relevant result consisted in demonstrating that two out of four of these antifungal DMPS (i.e., DMDiS and DMTriS) are taken up by Fg1 strain, which is evidenced with the observations of antifungal DMDiS in reduced quantity and even total depletion of the very antifungal DMTriS, in Fg1 cultures confronted to pure DMDiS and DMTriS. In the context of strong fungal growth inhibition by *C. vaccinii* bacterial VOCs against *Phoma* sp., another study reported a reduction, in dual-culture as compared to *C. vaccinii* monoculture, of DMTriS and 1-octanol, both identified as bacterial antifungal volatiles against *Phoma* sp. (65). Together with measurement of a total reduction of VOCs belonging to our *Microbacterium* strain, these results correlated positively the observation of VOCs depletion in dual-culture headspace with observation of VOCs antagonist bioactivity. This suggests a global uptake of bacterial bioactive VOCs by the growth-impacted fungus rather than a modulated production of bacterial VOCs in response to the interaction with the fungus. This uptake mechanism was verified with pure DMDiS and DMTriS antifungal VOCs. These results prove for the first time that the level of uptake of one VOC by the fungus could be a determinant feature explaining the intensity of its antifungal activity. Consequently, underlying key determinants of bacterial VOCs uptake, such as fungal wall adsorption or membrane auto-diffusion, could be a new approach for screening or identifying antifungal VOCs.

Nine VOCs were exclusively detected in dual-culture and are therefore co-culture induced. Among them are two fatty alcohol esters VOC subclass (acetic acid octyl ester and dimethyl octanol-1-acetate) and two fungal-attributed VOCs belonging to sesquiterpenes VOC subclass (gamma-muurolene, 7-epi-cis-sesquisabinene hydrate). Those sesquiterpenes are known to be produced by *Fusarium* fungi (66) and to have important diverse ecological functions including interactions with bacteria. We can conclude that those two VOCs act like signal molecules rather than being metabolic waste and their production to be activated by the interaction, maybe as a response to bacterial antifungal VOCs. These results are consistent with a previous study showing some VOCs are co-culture induced (65), proving the importance of feedback loops between fungal VOCs and bacterial VOCs resulting in the production of volatiles with potentially new properties.

Overall, these results indicate that volatiles produced by the antifungal *Microbacterium* and *Arthrobacter* rhizobacteria have the potential to be used to control the saprophytic wheat fungal pathogens *F. graminearum* and *Z. tritici*. To further evaluate the disease-suppressive efficacy of such volatiles in soils, it is necessary to in-depth study the metabolism of DMPS in soil. In a previous study, a DMPS-producing *Microbacterium* strain (45) was shown to bring similar plant growth promotion effects on *Arabidopsis* seedlings under *in vitro* and soil conditions. Similarly, biofumigation by soil incorporation of *Brassica* oil-less seed meals, which produce methanethiol, DMDiS, and DMTriS, showed direct toxic effects on pathogens including *F. oxysporum* (67), without adverse effects on plant development. These results support the hypothesis that DMPS are produced and diffused within the soil matrix, and could be active, under these conditions, against pathogens. The treatment of plant residues infected with *F. graminearum* and *Z. tritici* with DMPS-producing strains is of great interest, compared with the use of pure synthetic fumigants, such as DMDiS applied to soil surfaces. In fact, synthetic fumigants not only increase production costs, but also severely disrupt the soil ecosystem functioning. Indeed, repeated applications at high doses are often required to achieve the desired pest control. VOCs such as DMDiS or DMTriS are also toxic to a wide range of organisms, and large air emissions during fumigation pose serious environmental and human health concerns. In contrast, amendment of DMPS-producing strains into the rhizosphere can allow a localized and continuous production of bioactive volatiles while these strains are colonizing the rhizosphere and/or bulk soil. In addition, DMPS have a relatively high vapor pressure allowing their rapid dispersion in soils. Nevertheless, it is necessary to assess the impact of these VOC emissions on the total soil microbiota and in particular on beneficial fungi.

### Conclusion

To conclude, volatiles produced by *Microbacterium* and *Arthrobacter* wheat rhizosphere strains represent a new source of natural antifungal compounds, specifically DMPS, to control populations of challenging wheat pathogens: *F. graminearum* and *Z. tritici*. To further evaluate the biocontrol potential of these strains in soil and *in planta*, testing various inoculation methods would be a first step to determine the application of such strains. Our analysis of the volatilomics changes induced by the interaction between the *Microbacterium* antifungal strain and the *F. graminearum* pathogen allowed us to identify interaction-induced VOCs and to describe a high level of depletion of bacterial VOCs, further explained for both DMDiS and DMTriS antifungal VOCs by absorption mechanism by the pathogen’s mycelium. This knowledge advances our understanding of the underlying molecular mechanisms of volatile-mediated bacterial antagonism of DMPS against fungal pathogens and provides a basis for future experiments to validate the role of adsorption and/or absorption mechanisms in bacterial volatile-mediated antagonism on pathogen fungi.

## MATERIALS AND METHODS

### Bacterial and fungal strains and growth conditions

Five *Microbacterium* strains, namely *M. oxydans* JM188 and JM147.B, *M. foliorum* JM142, *M. yannicii* JM160, *M. schleiferi* JM177 and nine *Arthrobacter* strains, namely, *A. humicola* JM70, *A. globiformis* JM152, *A. aurescens* JM154.A, *A. phenanthrenivorans* JM84, and non-species determined *Arthrobacter* JM130, JM154.C, JM189, JM197, and JM201 were previously isolated from wheat (31). All strains were grown routinely on tryptone soy (TS medium; Carl Roth, Karlsruhe, Germany) for 48 h at 28°C. Long-term storage was done in 20% glycerol at −80°C.

The highly virulent French isolate *F. graminearum* MDC_Fg1 (referred to as *F. graminearum* Fg1) (68) and IPO323 *Z. tritici* isolate (69), collected on cultivar “Arminda” in the Netherlands in 1981, are the genome reference isolates used throughout experimentations.

*F. graminearum* Fg1 isolate was routinely grown on potato dextrose agar (PDA; Conda Pronadisa, Madrid, Spain) plates at 20°C in the dark for 8 days and subsequently stored at 4°C until required. IPO323 isolate was grown on yeast extract-peptone-dextrose medium (YPD) plates at 20°C in the dark for 7–10 days and subsequently stored at 4°C until required.

### Preparation of the *Z. tritici* spore suspension

For *in vitro* tests, spore suspensions of *Z. tritici* were created by transferring yeast-like spores from the YPD plate to 7 mL of YG medium (yeast extract-glucose). These were then incubated at 20°C in the dark for 10–15 days with stirring (120 rpm) after which spore concentrations were determined by counting on Thoma cell. A temperature of 20°C is the optimal temperature for spore production of *Z. tritici*. (70). A water suspension of 10^3^ spores/mL was prepared prior spreading on PDA plate for confrontation tests.

### *In vitro* dual culture assay for screening of the efficacy of rhizospheric strains and synthetic DMPS to inhibit the growth of *Fusarium graminearum and Zymoseptoria tritici*

The antifungal activity of the bacterial VOCs on both fungal pathogens was determined by measuring the mycelium growth with double--petri-dish setup. The bioassay system was set up with the bottoms of two lidless petri dishes (diameter, 85 mm), which were laid in opposition and then sealed together with two layers of parafilm and rubber band. The two parts of the dual culture were done as follow: one plate filled with PDA was either inoculated with a 6-mm plug of an 8 years old culture of *F. graminearum* Fg1 and placed in the center of the plate, or spread with 100 µL of a 10^2^ CFU/mL spore suspension of *Z. tritici* IPO323. The second petri dish, filled with TSA, was spread with 100 µL of bacterial suspension at 10^6^ CFU/mL. Bacterial suspensions were made inoculating bacteria cells from a 48-h TSA solid culture into TSB. Dual confrontation assay was fixed after 2 days of mycelium growth or directly after spores spreading of Z. *tritici,* and 1 day of bacterial growth at 28°C. The assembly was maintained with double layer of parafilm and rubber band. Three replicates per condition were conducted, and a same dual culture with spreading of TSB instead of bacterial inoculum on the bottom TSA plate was used as the negative control. Those dual cultures were incubated at 20°C in the dark.

To test the effects of synthetic sulfur volatile compounds dimethyl disulfide (DMDiS; Sigma-Aldrich Chimie, Saint-Quentin Fallavier, France) and dimethyl trisulfide (DMTriS; Sigma-Aldrich Chimie) and their mixture on the mycelium growth of the two pathogens, the same setting of dual culture was used with a petri dish containing the DMPS dilution, and the opposite one, the fungal culture. Synthetic DMPS were diluted in dimethylsulfoxide (DMSO; Euromedex, Mundolsheim, France) and added into a volume of 100 µL, reaching a total volumic concentration scaling from 11.7 to 352.5 nmol/cm^3^ for DMDiS and from 1.2 to 58.7 nmol/cm^3^ for DMTriS. Mixtures of the two DMDiS and DMTriS were tested using combinations of [DMDiS + DMTriS] equivalent to 11.7 + 1.2, 82.2 + 11.7, and 117.5 + 18.8 nmol*/*cm^3^. For controls, the second compartment was kept empty or 100 µL of DMSO was added. Petri dishes were immediately sealed and incubated.

Mycelium growth of *F. graminearum* Fg1 and *Z. tritici* IPO323 was pictured after respectively two and five days of contact with volatiles, and pictures were processed with ImageJ (71) to measure the growth areas. Growth inhibition was calculated relatively to DMSO control growth area. Three biological replicates were prepared and statistical differences were determined by one-way ANOVA with Tukey’s HSD test (*P* < 0.05).

### Sampling of volatiles from mono- or dual-culture assays with the *Microbacterium* and *Arthrobacter* strains in presence or absence of Fg1

In order to analyze the bacterial VOCs produced by the bacterial strains, an overnight bacterial culture of the strains in TSB was diluted at an OD_600_ of 0.1 and 100 µL were plated on sterile petri dishes containing 20 mL of TSA medium. Petri dishes were sealed with a double layer of parafilm and rubber band and incubated at 28°C for 3 days in order to allow the accumulation of VOCs before their collection. In addition, VOCs sampling from the dual-culture assays was also performed for JM188 + Fg1 confrontation and the associated controls, after 3 days of co-culture at 28°C. A solid-phase microextraction (SPME) was employed to collect headspace volatiles from the corresponding cultures using a polydimethylsiloxane-divinylbenzene (PDMS-DVB) fiber (Supelco, Bellefonte, PA, USA; 65 µm). The fiber was exposed to headspace volatiles for 60 min at 35°C prior to its removal, and analysis with a GC/MS system (see below). A total of three to four replicates per strain were done, and plates containing TSA medium only served as a control.

### Sampling of volatiles from dual-culture assays with the synthetic DMDiS and DMTriS in presence or absence of Fg1

In order to evidence the synthetic DMPS VOCs depletion by Fg1, a double-petri-dish setup was used, with one side containing 20 mL of PDA medium alone (control) or a 3-day Fg1 mycelium culture. The second side contained droplets of DMPS diluted in DMSO and added on a paper patch, with one of the two [DMDiS + DMTriS] combinations (82.2 + 11.7 or 0.94 + 0.47 nmol/cm^3^) being added into a volume of 100 µL. Dishes were sealed with a double layer of parafilm and rubber band and incubated at 20°C for 3 days before their collection using an SMPE fiber, for 60 min at 35°C, prior to their removal and analysis with a GC/MS system. A total of four replicates per condition were done. Correlation curves between area and DMPS volatile concentrations were obtained with a double-petri-dish setup with an empty dish on one side, and droplets of DMPS diluted in DMSO and added on a paper patch, with DMPS ranging from 0.09 to 94.4 nmol/cm^3^ for DMDiS and from 0.02 to 4.72 nmol/cm^3^ for DMTriS, on the other side. An incubation time of 1 h was used after sealing and VOCs were then sampled on SPME fibers, for 60 min at 35°C, prior to their removal and analysis with a GC/MS system.

### VOC profiling on culture headspace

Volatile compounds were analyzed by GC-QQQ-MS using a Hewlett Packard 7890A gas chromatograph (Agilent Technologies, Santa Clara, CA, USA) coupled to a triple quadrupole (QQQ) mass spectrometer (7000 A series, Agilent). A DB-5MS capillary column (60 m × 0.25 mm × 0.25 µm thick film; Agilent) was used to separate the VOCs. The temperature of the injection port was set at 250°C and a splitless mode was used. The column was heated using the following temperature program: the oven was held at 40°C for 2 min, then the temperature was raised to 270°C at a rate of 7°C/min, and the final temperature was held for 1 min. The helium carrier gas had a column flow rate of 2.3 mL/min. Mass spectra were acquired by electron impact ionization (70 eV) scanning from *m/z* 25 to 500 with a scan rate of 4 scans/s. The SPME-PDMS-DVB fiber desorption and cleaning were done during the first 30 min of the total heating program.

Detected peaks were manually integrated using MassHunter Qualitative Analysis B.07.00 software (Agilent Technologies). Alignment was done according to their retention time (RT) into a matrix using the GCalignR R package (72) and following parameters settings: max_diff_peak2peak = 0.02, min_diff_peak2peak = 0.08, max_linear_shift = 0.05. Only peaks found with areas at least two times higher than those found in sterile PDA and/or TSA media volatiles were kept in the analysis. Some alignments were manually adjusted. Compounds were tentatively identified comparing peaks mass spectra with those of the NIST (National Institute of Standards and Technology, USA) library (version 11.0) and by comparing the experimentally calculated linear retention indices (Kovats indices calculated with *n*-alcanes mix) with the literature values when available. Standards of DMDiS and DMTriS were analyzed and compared for retention time and mass spectra with identified pics from biological samples. To assess the capacity of the VOCs to discriminate the rhizobacteria according to their antagonistic levels, we performed a PCA using the R package FactoMineR (73) with two components and default parameters for the other options. After log10 transformation, a heatmap was made using the Pheatmap (74) package on areas of detected peaks over samples of dual-culture JM188 + Fg1 and corresponding controls. Hierarchical Ascendant Clustering over repetitions of the three conditions (rows of matrix) and each detected VOCs (column of the matrix) was performed using Euclidean distance-based clustering.

### DON and ADON mycotoxins extraction

*F. graminearum* Fg1 metabolite extraction was performed on fungal cultures (four replicates) corresponding to samples used for volatilomic analysis. Uninoculated PDA medium was also integrated as control condition. PDA agars, with or without fungal mycelium, were divided into small pieces of agar (5 mm × 5mm × 3 mm) and solid/liquid extraction was made with 20 mL of ethyl acetate per plate, with an incubation step during 10 min under agitation at 150 × *g,* followed by sonication for 10 min, centrifugation and recovery of the supernatant. The extraction protocol was repeated, giving a total extract volume of 40 mL per sample. Then, the organic phase (ethyl acetate) was dried using a SpeedVac (Centrivap Cold Trap Concentrator LABCONCO). Dried extracts were suspended into a calculated volume of methanol to reach 10 mg/mL.

### DON and ADON mycotoxins detection by liquid chromatography coupled with high-resolution mass spectrometry (LC-HRMS)

10 mg/mL of fungal extracts and mycotoxins standard solutions of the three DON, 15-ADON, and 3-ADON (diluted from 20 µg/mL to 500 ng/mL in methanol) were analyzed using Agilent Technologies Accurate-Mass Q-TOF LCMS 6530, with LC 1290 Infinity system. The separation was carried out at 40°C using a 120 EC-C18 column (3.0 × 100 mm × 2.7 µm; Agilent Poroshell). Elution gradient was the same as described by reference 75. Mass analyses were made in positive and negative mode, with the nebulization gas (Nitrogen) at a flow of 10 L/min and 40 psg pressure. The capillary tension was 3,000 V and gave ionization energy of 100 eV. Mycotoxin concentrations in the samples were calculated using standard curves correlating mycotoxins concentration to ion mass intensity, and linearity of correlation was verified from 0.5 to 20 µg/mL (*R*² > 0.996). Intensities of [M + COOH]^−^ ion were used for both DON and 3-ADON mycotoxins in MS−, and (M + H)^+^ ion for 15-ADON mycotoxin in MS+. Limit of detection for the three mycotoxins was measured as equal to 0.5 µg/mL.

## SUPPLEMENTAL MATERIAL

Supplemental figures spectrum.05292-22-s0001.pdf Fig. S1 to S3.

Table S1 spectrum.05292-22-s0002.xlsx List of VOCs from JM188 and Fg1 control and Fg1+JM188 confrontation conditions.

ASM does not own the copyrights to Supplemental Material that may be linked to, or accessed through, an article. The authors have granted ASM a non-exclusive, world-wide license to publish the Supplemental Material files. Please contact the corresponding author directly for reuse.
