# Supplementary material for: Dimethylpolysulfides production as the major mechanism behind wheat fungal pathogen biocontrol, by Arthrobacter and Microbacterium actinomycetes
Source: Microbiol Spectr. 2023 Oct 6;11(6):e05292-22. doi: 10.1128/spectrum.05292-22 (PMC10715130; doi:10.1128/spectrum.05292-22)
Supplement: Supplemental figures — Fig. S1 to S3. [file spectrum.05292-22-s0001.pdf]

SUPPLEMENTARY FIGURES

Dimethylpolysulfides production as the major mechanism behind wheat fungal pathogen-biocontrol, by *Arthrobacter* and *Microbacterium* actinomycetes

Aline Ballot[1], Jeanne Dore[1], Marjolaine Rey[1], Guillaume Meiffren[1], Thierry Langin[2], Pierre Joly[3*], Assia Dreux-Zigha [3], Ahmed Taibi[3], Claire Prigent-Combaret[1]

[1] *Laboratoire Ecologie Microbienne UMR 5557, Université Lyon 1, Villeurbanne, France*

[2] *Université Clermont Auvergne, INRAE, GDEC, Clermont-Ferrand, France.*

[3] *Greencell, Saint-Beauzire, France*

* *Vivagro, Technopole Montesquieu, 5 allée Jacques Latrille, 33650 Martillac*

Corresponding author : Claire Prigent-Combaret - claire.prigent-combaret@univ-lyon1.fr - UMR CNRS 5557 Ecologie Microbienne, Université Claude Bernard Lyon 1, 43 bd du 11 novembre 1918, F-69622 Villeurbanne, France.

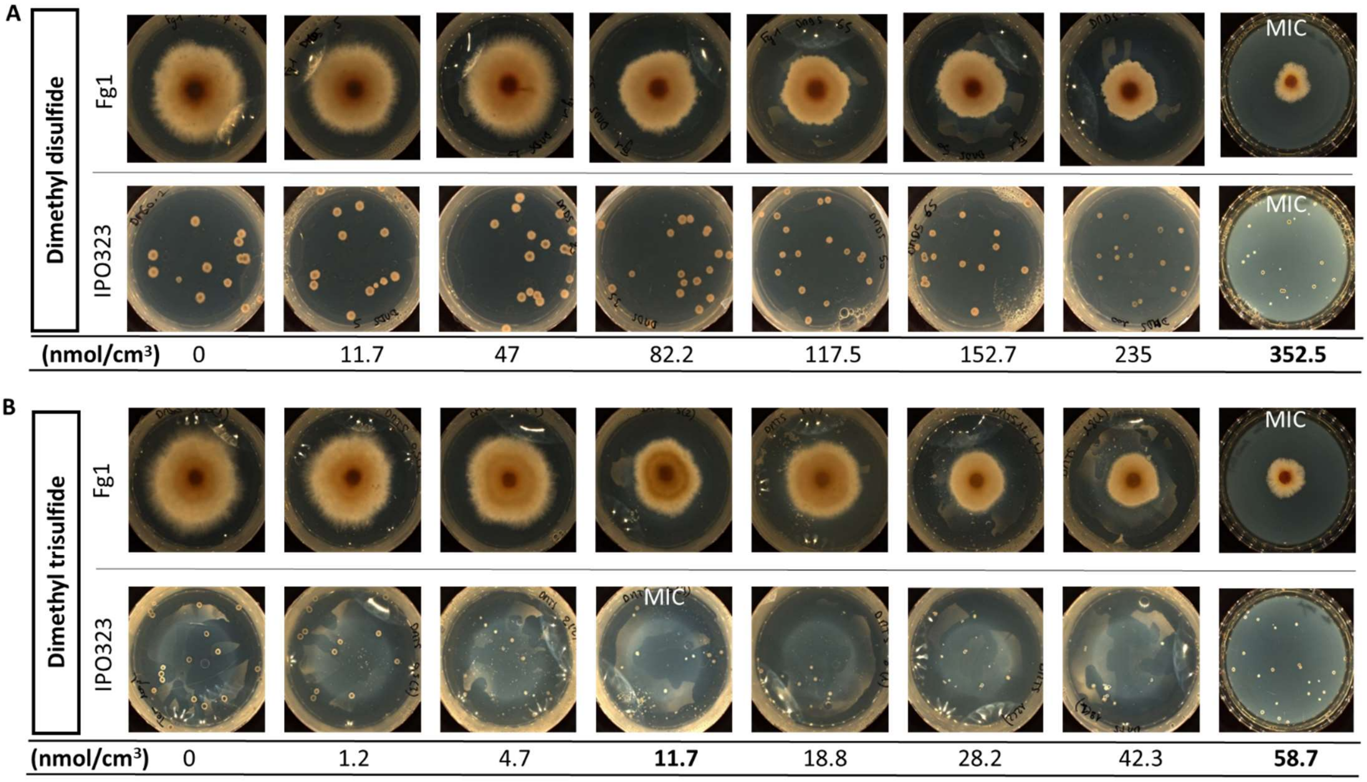

Fig S1. Mycelium growth of *F. graminearum* and *Z. tritici* exposed to gradual concentrations of (A) DMDiS (0 to 352.5 nmol/cm^3) or (B) DMTriS (0 to 58.7 nmol/cm^3). Pictures were taken 2 (*F. graminearum*) and 5 days (*Z. tritici*) after exposure. The Minimum Inhibitory Concentration (MIC) for each volatile x fungal pathogen is indicated in bold below the corresponding picture. The growth of the mycelium at 2 days, obtained before the confrontation, is similar to that observed for MIC values.

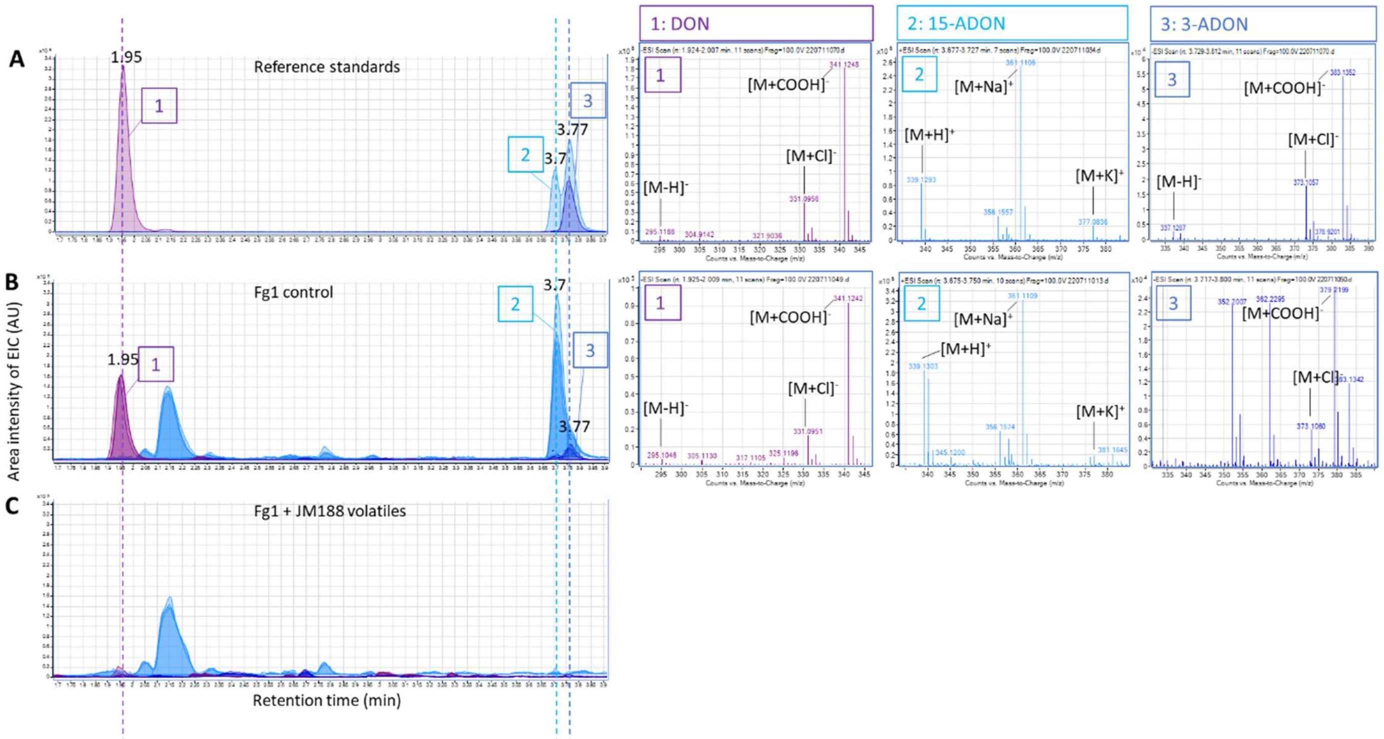

Fig S2. Extracted ion chromatograms (EIC) of DON and 15/3-ADON mycotoxins and associated mass spectra. (A) EIC over standard reference DON/ADON mixture (10µg/ml). (B) EIC over the four methanol extract replicates of Fg1 control condition. (C) EIC of the four methanol extract replicates of Fg1+ JM188. [M+COOH]$^-$ ion of DON mycotoxin is indicated at RT = 1.95, m/z= (1), [M+H]$^+$ ion of 15-ADON mycotoxin is indicated at RT = 3.7, m/z= (2) and [M+COOH]$^-$ ion of 3-ADON mycotoxin is indicated at RT = 3.77, m/z= (3).

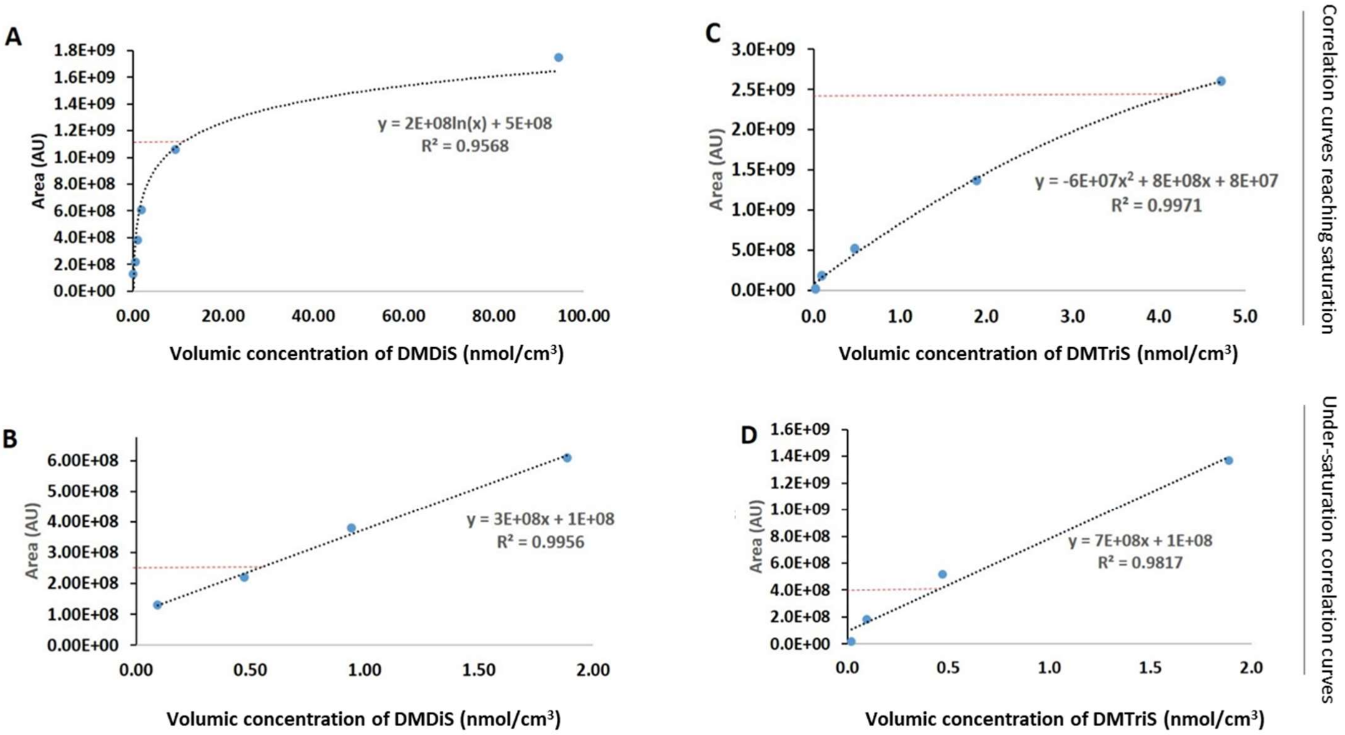

Fig S3. Correlation curves between area intensity *via* SPME/GC-MS and DMDiS and DMTriS concentrations introduced inside the double-Petri dishes setup. (A) Logarithmic correlation between area (AU) and volumic concentration (nmol/cm^3) of DMDiS, with concentrations ranging from 0.09 to 94.4 nmol/cm^3. Red spotted lateral bar indicates area mean value of detected DMDiS in the control condition without Fg1 culture, and in the presence of [82.2 DMDiS + 11.7 DMTriS] (nmol/cm^3) as presented in Fig. 7. (B) Linear-modeled correlation between area (AU) and volumic concentration (nmol/cm^3) of DMDiS, with concentration ranging from 0.09 to 1.9 nmol/cm^3. Red spotted lateral bar indicates area mean value of detected DMDiS in both control and Fg1 culture conditions, in the presence of [0.94 DMDiS + 0.47 DMTriS] (nmol/cm^3) as presented in Fig. 7. (C) Polynomnial correlation between area (AU) and volumic concentration (nmol/cm^3) of DMTriS, with concentration ranging from 0.02 to 4.72 nmol/cm^3. Red spotted lateral bar indicates area mean value of detected DMTriS in the control condition of Fg1 culture, in the presence of [82.2 DMDiS + 11.7 DMTriS] (nmol/cm^3) as presented in Fig. 7. (D) Linear-modeled correlation between area (AU) and volumic concentration (nmol/cm^3) of DMTriS, with concentration ranging from 0.02 to 1.9 nmol/cm^3. Red spotted lateral bar indicates area mean value of detected DMTriS in the control condition without Fg1 culture, in the presence of [0.94 DMDiS + 0.47 DMTriS] (nmol/cm^3) as presented in Fig. 7
